# PA3297 Counteracts Antimicrobial Effects of Azithromycin in *Pseudomonas aeruginosa*

**DOI:** 10.3389/fmicb.2016.00317

**Published:** 2016-03-16

**Authors:** Hao Tan, Lu Zhang, Yuding Weng, Ronghao Chen, Feng Zhu, Yongxin Jin, Zhihui Cheng, Shouguang Jin, Weihui Wu

**Affiliations:** ^1^State Key Laboratory of Medicinal Chemical Biology, Key Laboratory of Molecular Microbiology and Technology of the Ministry of Education, Department of Microbiology, College of Life Sciences, Nankai UniversityTianjin, China; ^2^Department of Molecular Genetics and Microbiology, College of Medicine, University of Florida, GainesvilleFL, USA

**Keywords:** RNA helicase, antibiotic resistance, azithromycin, rRNA processing, *Pseudomonas aeruginosa*

## Abstract

*Pseudomonas aeruginosa* causes acute and chronic infections in human. Its increasing resistance to antibiotics requires alternative treatments that are more effective than available strategies. Among the alternatives is the unconventional usage of conventional antibiotics, of which the macrolide antibiotic azithromycin (AZM) provides a paradigmatic example. AZM therapy is associated with a small but consistent improvement in respiratory function of cystic fibrosis patients suffering from chronic *P. aeruginosa* infection. Besides immunomodulating activities, AZM represses bacterial genes involved in virulence, quorum sensing, biofilm formation, and motility, all of which are due to stalling of ribosome and depletion of cellular tRNA pool. However, how *P. aeruginosa* responds to and counteracts the effects of AZM remain elusive. Here, we found that deficiency of PA3297, a gene encoding a DEAH-box helicase, intensified AZM-mediated bacterial killing, suppression of pyocyanin production and swarming motility, and hypersusceptibility to hydrogen peroxide. We demonstrated that expression of PA3297 is induced by the interaction between AZM and ribosome. Importantly, mutation of PA3297 resulted in elevated levels of unprocessed 23S-5S rRNA in the presence of AZM, which might lead to increased susceptibility to AZM-mediated effects. Our results revealed one of the bacterial responses in counteracting the detrimental effects of AZM.

## Introduction

*Pseudomonas aeruginosa* is a versatile Gram-negative pathogenic bacterium, that can cause various infections in human (de Bentzmann and Plésiat, 2011; Campa et al., 2012). During infection, *P. aeruginosa* produces multiple virulence factors to facilitate colonization (Sadikot et al., 2005; Kipnis et al., 2006; Hauser, 2009; Liu et al., 2015). Meanwhile, its highly intrinsic antibiotic resistance and biofilm forming ability greatly hinder the eradication of this pathogen (Høiby et al., 2005). In patients suffering from cystic fibrosis (CF) and chronic obstructive pulmonary disease (COPD), *P. aeruginosa* caused chronic respiratory infections are responsible for most of the morbidity and mortality (Rabin et al., 2004; Rada and Leto, 2013). Intensive antibiotic treatment has been used to maintain the lung function and extend lifespan of the patients (Doring et al., 2000). However, the increasing antibiotic resistance has been compromising clinical efficacy of traditional antibiotics. Thus, alternatives or unconventional usage of the antibiotics are urgently needed (Breidenstein et al., 2011; Poole, 2011; Imperi et al., 2014).

The macrolide antibiotic azithromycin (AZM) provides a paradigmatic example of an unconventional antibacterial drug for *P. aeruginosa* treatment. Although, *P. aeruginosa* is highly resistance to macrolides owning to its low outer membrane permeability and the resistance-nodulation-cell division (RND) systems, AZM treatment benefits patients suffering from both intermittent and chronic *P. aeruginosa* infections (Saiman et al., 2003; Lister et al., 2009; Blasi et al., 2010; Steel et al., 2012; Aminov, 2013; Morita et al., 2013). AZM has been shown to have immunomodulatory activity, which attenuates the inflammatory response and promotes macrophage phagocytic activity (Legssyer et al., 2006; Steinkamp et al., 2008; Tsai et al., 2009). Furthermore, AZM exhibits bactericidal effect on stationary growth phase *P. aeruginosa* cells (Lovmar et al., 2004, 2009; Imamura et al., 2005; Köhler et al., 2007; Starosta et al., 2010; Gödeke et al., 2013). And sub-inhibitory concentrations of AZM suppress biofilm formation, motility, and production of multiple virulence factors, including proteases, pyocyanin, exotoxin A, phospholipase C (PLC), exopolysaccharides, and other quorum-sensing (QS) regulated genes in *P. aeruginosa* (Molinari et al., 1992, 1993; Tateda et al., 2001; Favre-Bonté et al., 2003; Gillis and Iglewski, 2004). The AZM-mediated killing of stationary-phase bacterial cells and reduced expression of QS-regulated virulence factors require interaction between AZM and ribosome (Köhler et al., 2007). AZM binds in the nascent peptide exit tunnel (NPET), resulting in ribosome stalling and depletion of the intracellular pools of aminoacyl-tRNAs (Lovmar et al., 2004, 2009; Köhler et al., 2007; Starosta et al., 2010; Gödeke et al., 2013). The effects of AZM on *P. aeruginosa* can be counteracted by over expression of ErmBP or a peptidyl-tRNA hydrolase, which blocks the interaction between AZM and ribosome by modifying the 23S rRNA or increases the intracellular aminoacyl-tRNA level, respectively (Köhler et al., 2007; Gödeke et al., 2013). However, how *P. aeruginosa* response to AZM treatment remains unclear. Understanding the mechanisms that *P. aeruginosa* uses to counteract AZM treatment may provide clues to enhance AZM-mediated virulence inhibitory and bacterial killing effects.

A large RNA helicase family named DExD/H box helicases are characterized by a conserved DExD/H box sequence (Cordin et al., 2006; Linder and Jankowsky, 2011), and play crucial roles in rRNA processing, translation initiation, and mRNA decay (Iost et al., 2013; Linder and Fuller-Pace, 2013). In addition, the DExD/H box helicases have been shown to participate in bacterial responses to various stresses, such as cold shock, pH, osmotic, and oxidative stresses (Owttrim, 2013). And several DEAD family RNA helicases, which belong to a specific subfamily of DExD/H box helicases, have been shown to regulate virulence factors in *Escherichia coli*, *Borrelia burgdorferi*, *Staphylococcus aureus*, *Listeria monocytogenes*, and *P. aeruginosa* (Koo et al., 2004; Salman-Dilgimen et al., 2011, 2013; Oun et al., 2013; Bareclev et al., 2014; Intile et al., 2015). The pleiotropic functions of DExD/H box family RNA helicases intrigued us to suspect that they might be involved in the bacterial response to AZM treatment. In this study, we found that deficiency in a DEAH box helicase, PA3297, renders *P. aeruginosa* more susceptible to the killing and virulence suppression by AZM. Our results suggest that the expression of PA3297 was up regulated in the presence of AZM, which might promote 23S rRNA maturation to counteract the inhibitory effect of AZM on protein elongation.

## Materials and Methods

### Strains and Plasmids

The bacterial strains and plasmids used in this study are listed in **Table 1** (Simon et al., 1983; Hoang et al., 1998; Choi and Schweizer, 2006; Liberati et al., 2006). The *E. coli* strains DH5α, S17-1 and *P. aeruginosa* strains were routinely cultured in Luria-Bertani (LB) broth at 37°C. Antibiotics were used at the following concentrations: for *E. coli*, ampicillin 100 μg/ml, tetracycline 10 μg/ml, and gentamicin 10 μg/ml; for *P. aeruginosa*, carbenicillin 150 μg/ml, tetracycline 50 μg/ml, and gentamicin 50 μg/ml.

**Table 1:** Bacterial strains and plasmids used in this study.

Strain or plasmid	Description^a^	Reference or origin
***E. coli* strains**		
DH5α	F^-^, φ80d*lacZ*Δ*M15*,Δ*(lacZYA-argF)U169, deoR, recA1, endA1, hsdR17(r_k_*^-^*,m_k_^+^), phoA, supE44, λ*^-^*, thi1, gyrA96, relA1*	TransGen
S17-1	*thi pro hsdR recA* Tra^+^	Simon et al., 1983
***P. aeruginosa* strains**		
PA14	Wild type *Pseudomonas aeruginosa* strain PA14	Liberati et al., 2006
PA0426::Tn	PA14 with a transposon inserted at PA0426	Liberati et al., 2006
PA0455::Tn	PA14 with a transposon inserted at PA0455	Liberati et al., 2006
PA2840::Tn	PA14 with a transposon inserted at PA2840	Liberati et al., 2006
PA3002::Tn	PA14 with a transposon inserted at PA3002	Liberati et al., 2006
PA3272::Tn	PA14 with a transposon inserted at PA3272	Liberati et al., 2006
PA3297::Tn	PA14 with a transposon inserted at PA3297	Liberati et al., 2006
PA3308::Tn	PA14 with a transposon inserted at PA3308	Liberati et al., 2006
PA3861::Tn	PA14 with a transposon inserted at PA3861	Liberati et al., 2006
PA3950::Tn	PA14 with a transposon inserted at PA3950	Liberati et al., 2006
ΔPA3297	PA14 with PA3297 in frame deletion	This study
ΔPA3297/att7::PA3297	PA14 ΔPA3297 with insertion of a single copy of PA3297 driven by its own promoter at attTn7 sites	This study
ΔPA3297/att7::PA3297 K101A	PA14 ΔPA3297 complemented with a single copy of PA3297 acquired a lysine101 mutation to alanine	This study
ΔPA3297/att7::PA3297 D192A	PA14 ΔPA3297 complemented with a single copy of PA3297 acquired an aspartate192 mutation to alanine	This study
ΔPA3297/att7::PA3297SAT224AAA	PA14 ΔPA3297 complemented with a single copy of PA3297 acquired a serine224 and threonine226 mutation to alanines	This study
**Plasmids**		
pEX18Tc	Broad-host-range gene replacement vector; *sacB* TET^r^	Hoang et al., 1998
pUC18t-mini-Tn7T-Gm	For gene insertion in chromosome; GEN^r^	Choi and Schweizer, 2006
pTNS3	Helper plasmid	Choi and Schweizer, 2006
pFLP2	Source of Flp recombinase; *sacB*, AMP^r^/CAR^r^	Hoang et al., 1998
pTH1501	pEX18Tc::ΔPA3297; TET^r^	This study
pTH1502	PA3297 gene of PA14 on pUC18T-Mini-Tn7T-Gm with its own promoter; GEN^r^	This study


*^a^AMP^*r*^, ampicillin resistant; TET^*r*^, tetracycline resistant; GEN^*r*^, gentamicin resistance; CAR^*r*^, carbenicillin resistant.*

### DNA Methods

DNA manipulations were performed according to standard protocols or following manufacturers’ instructions (Hoang et al., 1998; Zheng et al., 2004; Choi and Schweizer, 2006). The pEX18Tc::ΔPA3297 (pTH1501) was constructed by cloning the 1002-bp upstream and 964-bp downstream fragments of PA3297 coding region into the KpnI-HindIII sites of plasmid pEX18Tc. The fragments were amplified from the PA14 chromosome with primers PA14-UPA3297-FF, PA14-UPA3297-FR, PA14-DPA3297-FF, and PA14-DPA3297-FR (**Table 2**), respectively. Deletion of the PA3297 gene was confirmed by PCR with primers PA14-PA3297-FF and PA14-PA3297-FR (**Table 2**). For the complementation of PA3297, the PA3297 gene was amplified from the PA14 chromosome by PCR with the primers PA14-PA3297-FF and PA14-PA3297-FR (**Table 2**). The PCR product was ligated into the EcoRI- SacI sites of pUC18t-mini-Tn7T-Gm, resulting in pTH1502. The plasmid was introduced into the ΔPA3297 mutant by electroporation, along with the helper plasmid pTNS3 (Choi and Schweizer, 2006). Insertion of the PA3297 gene into the chromosome was confirmed by PCR with primers P_Tn7R_ and P_glmS-down_ (**Table 2**; Choi and Schweizer, 2006). The site-directed mutagenesis was performed as previously described (Zheng et al., 2004). The mutation sites were chosen based on the conserved critical residues of other bacterial DExD-box proteins (Koo et al., 2004; Cordin et al., 2006). Briefly, PCR amplification was performed with pTH1502 as template and with primers listed in **Table 2**, for K101A, D192A, and SAT224-226AAA mutations, respectively. The PCR products were treated by DpnI for 3 h at 37°C and purified before transformation. The correctly mutated clones were identified by DNA sequencing.

**Table 2:** Primers used in this study.

Primers^a^	Nucleotide sequence (53)^b^
**Cloning of upstream and downstream fragments for PA3297 deletion**	
PA14-UPA3297-FF	GAAAGCGGTACCGAAGTAAGTCCGCCGTTGCC (Kpn I)
PA14-UPA3297-FR	CAGCTTTCTAGAGGTGCTGTCGTCGCTCTGGT (Xba I)
PA14-DPA3297-FF	TTGCAGTCTAGACGCTGGATGCTGGAGGAGTA (Xba I)
PA14-DPA3297-FR	CGCCGGAAGCTTCACCGAGCAGTGGCTGAAGAC (Hind III)
**Cloning of gene PA3297 for complementation**	
PA14-PA3297-FF	TGAAGAGAATTCGCCAGAAGTAAGTCCGCCGTTGCC (EcoR I)
PA14-PA3297-FR	CACCGGGAGCTCCGACCAGACCGACCTGTTCTTCACCAT (Sac I)
P_Tn7R_	CACAGCATAACTGGACTGATTTC
P_glmS-down_	GCACATCGGCGACGTGCTCTC
**Primers used for site-directed mutagenesis of PA3297**	
PA3297-K101A-FF	GCGAGACCGGCTCGGGC 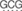 ACCACCCAG
PA3297-K101A-FR	T 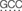 GCCCGAGCCGGTCTCGCCGGCGATC
PA3297-D192A-FF	TACGACACGCTGATCGTC 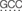 GAAGCCCAC
PA3297-D192A-FR	C 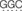 GACGATCAGCGTGTCGTAGCGCTCCAG
PA3297-SAT224-226AAA-FF	GCTGATCATCACC 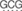 GCG 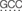 ATCGACCTGGAG
PA3297-SAT224-226AAA-FR	T 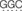 CGC 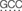 GGTGATGATCAGCTTCAGGTCC
**RT-qPCR primers for gene expression measurements**	
23S-R-FF	AAAGATAACCGCTGAAAG
23S-R-FR	CTATCAACGTCGTAGTCT
5S-R-FF	CGAACTCAGAAGTGAAAC
5S-R-FR	CTTGACGATGACCTACTC
23S-5S-R-FF	GTACTAATTGCCCGTGAG
23S-5S-R-FR	GTTCCAACGCTCTATGAT


*^a^The direction of the primer is indicated at the end of the primer designation as follows: FF for forward and FR for reverse. ^b^The solid underlines are the sites of listed restriction enzymes; the dotted underlines are the sites for mutagenesis, with all the residues changed into alanine.*

### Assay for Pyocyanin Production

The pyocyanin concentration was determined as described previously (Essar et al., 1990). Briefly, 1 ml supernatant from each 24-h-old bacteria culture grown in the absence or presence of AZM was extracted with 0.5 ml of chloroform. Then, 0.4 ml solution from the lower organic phase was re-extracted into 0.3 ml of 0.2 N HCl to give a pink solution, whose absorbance was measured at 520 nm. Concentrations of pyocyanin (mg/ml) were calculated by multiplying the OD_520_ by 32.01 (Kurachi, 1958).

### Antibiotic Susceptibility Assay

Minimum inhibitory concentrations (MICs) of *P. aeruginosa* to antimicrobial agents were determined by serial twofold broth dilution in LB medium, as described previously (Jo et al., 2003). MICs were recorded as the lowest concentration of antibiotic inhibiting visible growth after 24 h of incubation at 37°C.

### Stationary-Phase Bacterial Cell Killing Assay

The killing assay was performed as described previously (Köhler et al., 2007). Briefly, bacteria were inoculated in LB medium and grown for 16 h at 37°C. The culture of each strain was diluted to an OD_600_ of 0.05 and cultured at 37°C. After reaching stationary phase (OD_600_, 3.0), indicated concentrations of AZM were added to the 2-ml aliquots of the cultures. Then the bacteria were cultured for 20–22 h at 37°C. The viable bacterial numbers were determined by serial dilution and plating on drug-free LB agar plates. The survival rate of each strain was calculated as live bacterial number in AZM treated sample divided by the bacterial number of the corresponding untreated sample.

### Biofilm Tolerance to AZM

The biofilm resistance was measured as previously described with minor modifications (Bjerkan et al., 2009; Billings et al., 2013; Liao et al., 2013). Briefly, overnight bacterial cultures were diluted to an OD_600_ of 0.025. 150 μl of the bacteria were incubated in each well of a 96-well plate at 37°C without agitation (Brencic et al., 2009). After 24 h, the planktonic bacteria were discarded by aspiration. Then, the biofilms were treated with 150 μl LB medium containing indicated concentrations of AZM for 2 h. The medium in each well was replaced with fresh LB medium, and subjected to sonication at a frequency of 40 kHz, with a power output of 300 W, at 37°C for 5 min. The live bacteria were enumerated by serial dilution and plating.

### Growth Assay

Overnight culture of each strain was diluted into fresh LB (150 μl) to an OD_600_ of 0.05 in each well of a 96-well plate without or with different concentrations of AZM. The plate was incubated at 37°C with constant agitation (Lau et al., 2012; Guénard et al., 2014). The bacterial growth was monitored by measuring the OD_600_ every 30 min for 12 h by a Varioskan Flash microplate reader (Thermo Electron Corporation).

### H_2_O_2_ Susceptibility Assay

Overnight cultures of the *P. aeruginosa* strains were diluted to an OD_600_ of 0.05 and cultured at 37°C. When the OD_600_ reached 0.3 (about 1.5–2 h later), AZM (0.5 μg/ml) was added if needed. When the OD_600_ reached 2.0 (about 3 h later), bacteria from 500 μl culture were collected and washed twice with phosphate buffered saline (PBS). Then the bacteria were resuspended in PBS with or without 10% H_2_O_2_ and incubated for 15 min. The live bacterial numbers were determined by serial dilution and plating.

### Motility Assay

The swarming motility was tested on modified M9 medium plates supplemented with 0.2% glucose, 1 mM MgSO_4_, and 0.05% glutamate as the nitrogen source. 0.5% agar was used for solidification. Two microliters of exponential growth phase *P. aeruginosa* was deposited on the plates, then incubated for 18 h at 37°C (Köhler et al., 2007).

### Total RNA Isolation and Quantitative Real-Time PCR

Overnight cultures of *P. aeruginosa* strain PA14 and ΔPA3297 were diluted into fresh LB medium to an OD_600_ of 0.05. The bacteria were grown at 37°C with agitation (200 rpm). When the OD_600_ reached 0.3 (about 1.5–2 h later), AZM was added to reach indicated concentrations. Samples were harvested when the OD_600_ reached 2.0 (about 3 h later; Skindersoe et al., 2008). Total RNA was isolated with an RNeasy Minikit (Tiangen Biotech). The cDNA from each RNA sample was synthesized with reverse transcriptase and random primers (Takara). Real-time PCR was performed with SYBR premix Ex Taq (Roche). The conserved hypothetical protein coding gene PA1769 was used as an internal control (Son et al., 2007). The primers used in quantitative real-time PCR were listed in **Table 2**, with a designation of “RT-qPCR.”

### Statistical Analysis

When indicated, Student’s *t*-test (two-tailed) was used to determine whether the deletion of PA3297 resulted in any significant differences compared to the wild-type cells treated with the same concentrations of AZM.

## Results

### Deficiency of PA3297 Intensifies the Effects of AZM on Pyocyanin Production

**FIGURE 1:**
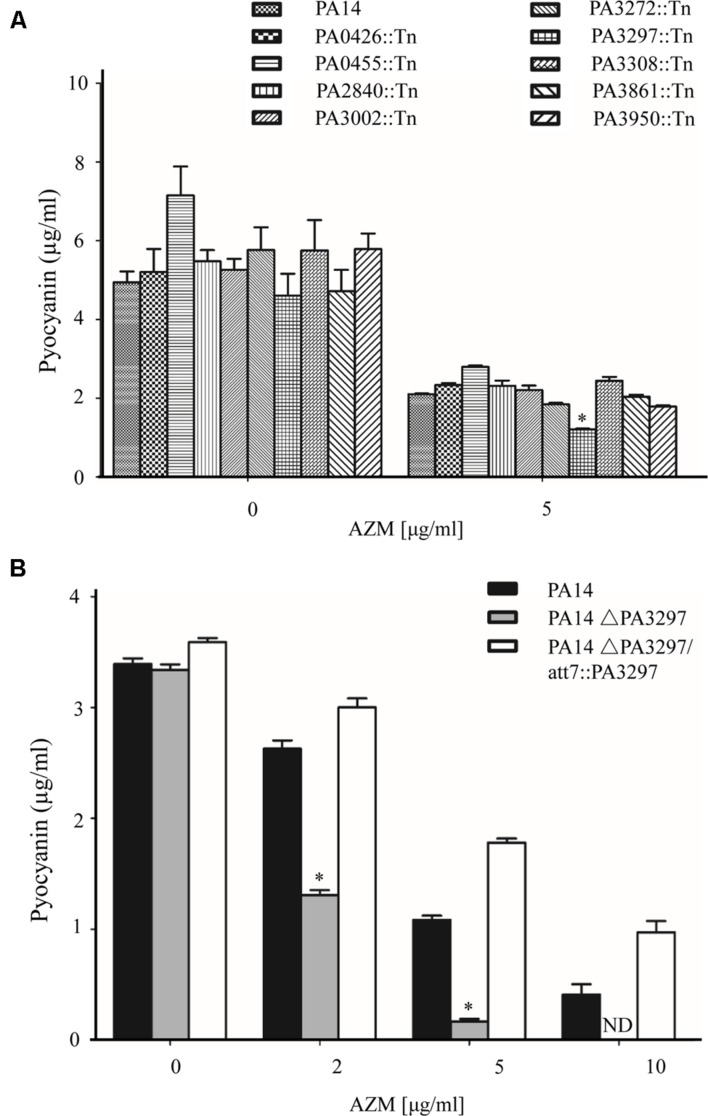
**Mutation of PA3297 intensified the AZM mediated inhibition of pyocyanin production. (A)** Wild type PA14 and the mutants of DExD/H box helicases were grown to an OD_600_ of 2.0 in the absence or presence of 5 μg/ml AZM at 37°C and the pyocyanin concentrations were measured. **(B)** Bacteria of wild type PA14, ΔPA3297 mutant and the complementation strain were grown in the absence or presence of 2, 5, or 10 μg/ml AZM. When the OD_600_ reached 2.0, the pyocyanin concentrations were measured. The values are the means of three replicates and the error bars display the standard deviations. “ND” standards for “not detected.” ^∗^*p* < 0.05 compared to PA14 or the complemented strain by student’s *t*-test.

As pyocanin production is suppressed by AZM (Molinari et al., 1992, 1993; Tateda et al., 2001; Favre-Bonté et al., 2003; Gillis and Iglewski, 2004), which can be easily observed and quantified, we used this phenotype to test whether DExD/H box RNA helicases are involved in bacterial response to AZM treatment. There are 17 DExD/H box RNA helicases in the genome of *P. aeruginosa* strain PA14 (www.pseudomonas.com; Winsor et al., 2011). There are nine DExD/H box helicase mutants in the non-redundant PA14 transposon mutants library, however, the other eight DExD/H box helicase mutants are not available (**Table 1**; Liberati et al., 2006; Breidenstein et al., 2011). Thus, we examined pyocyanin production of the available mutants in the absence and presence of AZM at an OD_600_ of 2.0. A PA3297::Tn mutant displayed a significant decrease in pyocyanin production in the presence of AZM, whereas no difference was observed in the absence of AZM (**Figure 1A**). To confirm the role of PA3297, we generated an in frame deletion of PA3297 in PA14. The wild type PA14 and the ΔPA3297 mutant were grown in the absence or presence of 2, 5, or 10 μg/ml AZM. When the bacteria reached same density (OD_600_ of 2.0), we measured the pyocyanin levels. AZM inhibited the production of pyocyanin in the wild type strain in a dose dependent manner. However, the production of pyocyanin by the mutant was repressed more severely in the presence of AZM at all the tested concentrations (**Figure 1B**). Complementation with a PA3297 gene restored the production of pyocyanin (**Figure 1B**).

### Increased Killing of the ΔPA3297 Mutant by AZM

**FIGURE 2:**
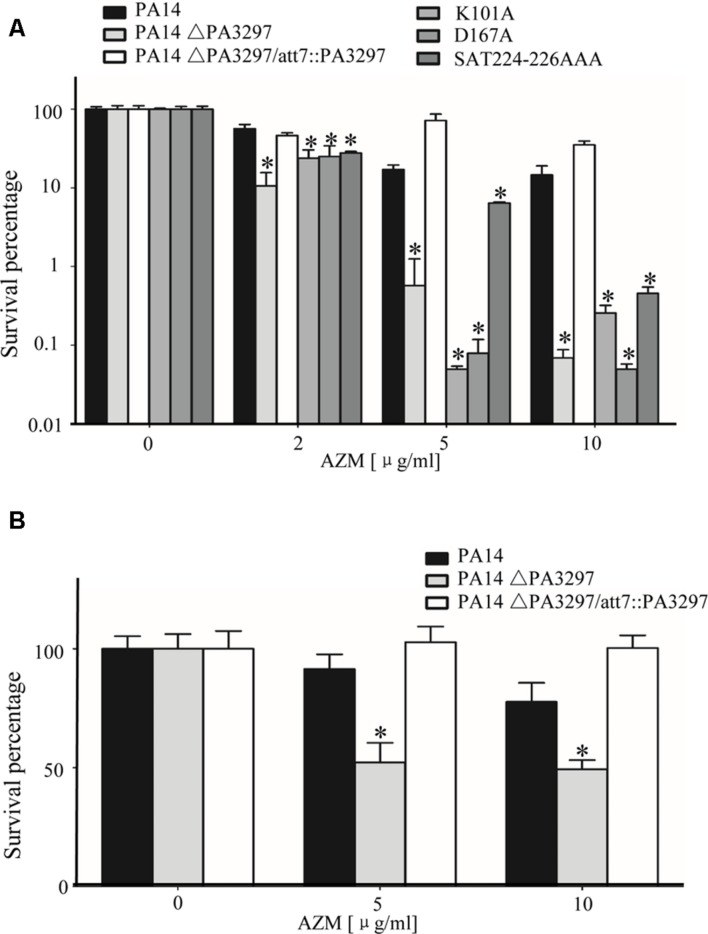
**Increased killing of the ΔPA3297 mutant by AZM. (A)** AZM-mediated killing of wild type PA14, the ΔPA3297 mutant and the mutant complemented with wild type or the mutated PA3297. Stationary-phase bacteria were treated with 2, 5, or 10 μg/ml AZM for 20 h at 37°C. The numbers of live bacteria were determined by serial dilution and plating. **(B)** AZM-mediated killing of biofilm. 24-h-old biofilms formed by wild type PA14, the ΔPA3297 mutant and the complemented strain were treated with 5 μg/ml, 10 μg/ml AZM or not for 2 h. Bacteria in the biofilm were dissociated from the wells by gentle sonication. The viable bacteria were determined by serial dilution and plating. The averages and associated standard deviations from three replicates are shown. ^∗^*p* < 0.05 compared to PA14 or the complemented strain by student’s *t*-test.

Stationary growth phase *P. aeruginosa* cells are susceptible to AZM (Lovmar et al., 2004, 2009; Imamura et al., 2005; Köhler et al., 2007; Starosta et al., 2010; Gödeke et al., 2013). To test whether mutation of PA3297 renders higher susceptibility, we performed the stationary-phase cells killing assay as previously described (Imamura et al., 2005; Köhler et al., 2007; Gödeke et al., 2013). Stationary-phase cells of PA14, the ΔPA3297 mutant and the complementation strain were subjected to treatment with AZM at the concentrations of 2, 5, and 10 μg/ml. As shown in **Figure 2A**, the AZM-mediated killing of the ΔPA3297 mutant was significantly increased at all of the AZM concentrations tested. The most significant difference was observed at the concentration of 10 μg/ml, where the survival rate of the mutant was approximately 1% of those of the wild type and complemented strains. In addition, the ΔPA3297 mutant was more susceptible to another macrolid antibiotic, erythromycin, with a fourfold lower MIC compared to the wild type strain PA14 (**Table 3**).

**Table 3:** Bacterial susceptibilities to macrolides and lincosamides.

Strain	MIC (μg/ml)^a^			
	
	ERY	AZM	LIN	CLI
PA14	300	400	12800	4800
ΔPA3297	75	100	3200	2400
ΔPA3297/att7::PA3297	300	400	>12800	9600
ΔPA3297/att7::PA3297 K101A^b^	75	100	6400	1600
ΔPA3297/att7::PA3297 D192A^b^	75	100	3200	2400
ΔPA3297/att7::PA3297 SAT224AAA^b^	75	100	6400	2400


*^a^ERY, erythromycin; AZM, azithromycin; LIN, lincomycin; CLI, clindamycin.*

*^b^Mutants with single or triple amino acid changes.*

Next, we tested the susceptibility of the ΔPA3297 mutant to lincosamides, whose bactericidal mechanism is similar to macrolides (Tenson et al., 2003; Wilson, 2014). Indeed, mutation in PA3297 increased the bacterial susceptibility to lincomycin and clindamycin (**Table 3**). However, no increase of susceptibility was observed to other antibiotics, including ciprofloxacin, carbenicillin, meropenem, tetracycline, tobramycin, kanamycin, chloramphenicol, or polymyxin B (**Supplementary Table S1**). These results suggest that PA3297 plays an important role in the resistance against antibiotics targeting the peptide exit tunnel of ribosome.

PA3297 is a putative RNA helicase belonging to the DEAH-box family proteins, which are characterized by the presence of seven to nine conserved motifs (Tanner and Linder, 2001). In the *E. coli* RNA helicases DbpA and HrpA, the conserved residues GETGSGKT in motifI, DEAH in motifII, and SAT in motifIII have been shown to be required for interaction with and hydrolysis of NTP (Koo et al., 2004; Linder and Fuller-Pace, 2013). To determine whether these critical residues within motifs I, II, and III are important for PA3297 in the resistance to macrolides and lincosamides, we altered the residues by site-directed mutagenesis. Specifically, the K101 in motif I or the D167 in motif II was mutated to alanine. The S224 and T226 in motif III were both replaced with alanine. Each mutated PA3297 was transferred into the ΔPA3297 mutant and the susceptibility to antibiotics was tested. None of the mutated PA3297 was able to restore the survival rate of the mutant (**Figure 2A**). In addition, the mutated PA3297 was unable to restore the resistance of the ΔPA3297 mutant to macrolides, lincomycin, and clindamycin (**Table 3**). These results suggest that the RNA helicase function of PA3297 is required for its role in the resistance to macrolides and lincosamides.

Clinically, AZM has been used in the treatment of chronic *P. aeruginosa* infection (Saiman et al., 2003; Blasi et al., 2010), which is characterized by biofilm formation (Singh et al., 2000). The biofilm is notorious for high antibiotic tolerance, which severely hinders eradication of the bacteria (López et al., 2010; Breidenstein et al., 2011). We suspected that mutation of PA3297 might increase the killing efficacy of AZM on the biofilm. Indeed, the survival rates of the ΔPA3297 mutant in biofilm were lower than those of the wild type PA14 at various AZM concentrations, which were restored by complementation with a wild type PA3297 (**Figure 2B**).

### Mutation of PA3297 Increases the Bacterial Susceptibility to Hydrogen Peroxide in the Presence of AZM

**FIGURE 3:**
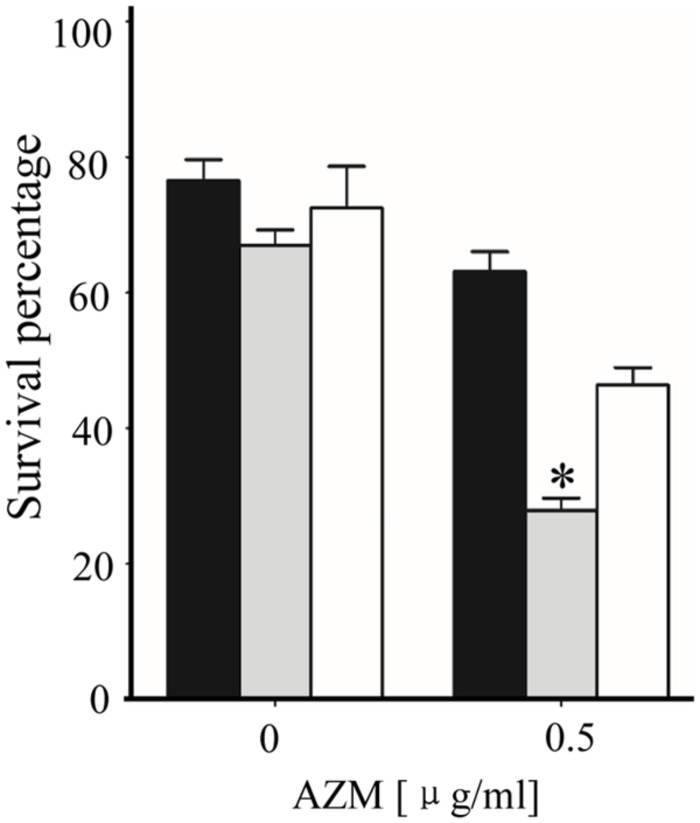
**Hydrogen peroxide (H_2_O_2_) mediated killing of bacteria.** PA14, the ΔPA3297 mutant and complemented strain were grown at 37°C in the absence or presence of 0.5 μg/ml AZM to an OD_600_ of 2.0. Bacteria were collected and washed with PBS. Then the bacteria were incubated in PBS with or without 10% H_2_O_2_ for 15 min. The live bacterial numbers were determined by serial dilution and plating. ^∗^*p* < 0.05 compared to PA14 or the complemented strain by student’s *t*-test.

It has been shown that AZM treatment impairs the oxidative stress response in *P. aeruginosa* (Nalca et al., 2006), which intrigued us to test whether mutation of PA3297 leads to further impairment. The H_2_O_2_ susceptibility assay was performed in the presence of 0.5 μg/ml AZM, as the growth rates of wild type PA14 and the ΔPA3297 mutant were similar at this concentration of AZM (**Supplementary Figure S1**). The wild type PA14, ΔPA3297 mutant and the complemented strain were grown without or with AZM to an OD_600_ of 2.0, and then treated with 10% H_2_O_2_ for 15 min. In the presence of AZM, the ΔPA3297 mutant was more susceptible to H_2_O_2_ than the wild type and complemented strains, whereas no difference was observed in the absence of AZM (**Figure 3**). These results suggest that PA3297 is involved in the bacterial oxidative stress response in the presence of AZM.

### Mutation of PA3297 Intensifies the Inhibitory Effect of AZM on Swarming Motility

**FIGURE 4:**
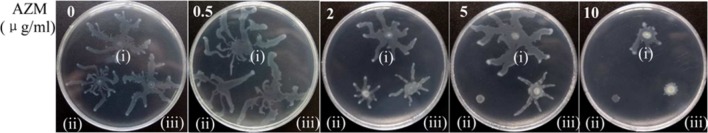
**Bacterial swarming motility.** PA14 (i), the ΔPA3297 mutant (ii) and the complemented strain (iii) were inoculated on plates containing indicated concentrations of AZM.

Besides oxidative stress response, AZM suppresses swarming motility (Tateda et al., 2001; Köhler et al., 2007; Gödeke et al., 2013). Same numbers of wild type PA14 and the ΔPA3297 mutant were inoculated on the plates containing various concentrations of AZM. As shown in **Figure 4**, 10 μg/ml AZM suppressed the swarming motility of wild type PA14 obviously, whereas the lower concentrations of AZM showed no inhibitory effect. However, starting from 2 μg/ml, AZM suppressed the swarming motility of the ΔPA3297 mutant in a dose dependent manner. Complementation with a PA3297 gene restored the swarming motility in the presence of AZM (**Figure 4**).

### Interaction between AZM and Ribosome Induces the Expression of PA3297

**FIGURE 5:**
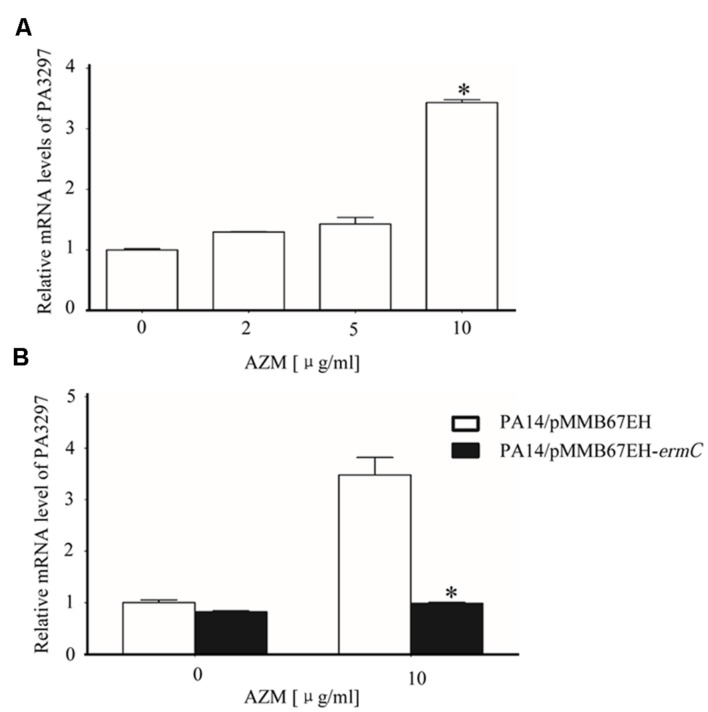
**Expression of PA3297 in the presence of AZM. (A)** Wild type PA14 was grown to an OD_600_ of 0.3 in LB medium. The bacteria were grown further in the absence or presence of 2, 5, or 10 μg/ml AZM. When the OD_600_ reached 2.0, total bacterial RNA was isolated and the mRNA levels of PA3297 were determined with real time PCR. ^∗^*p* < 0.05 compared to bacteria in the absence of presence of 2 or 5 μg/ml AZM by student’s *t*-test. **(B)** PA14 harboring pMMB67EH or the ErmC over expressing plasmid (pMMB67EH-*ermC*) was grown at 37°Cin the absence of AZM. When the OD_600_ reached 0.3, 1 mM IPTG was added to the medium. Meanwhile, no AZM or AZM at the final concentration of 10 μg/ml was added to the medium. At the OD_600_ of 2.0, total RNA was isolated and the mRNA levels of PA3297 were determined with real time PCR. The mRNA levels of *lacI* from the plasmid were used as internal control. ^∗^*p* < 0.05 compared to PA14/pMMB67EH by student’s *t*-test.

Our results so far suggested that PA3297 is involved in the bacterial response to AZM treatment. To test whether the expression of PA3297 is induced by AZM treatment, wild type PA14 was grown in the absence or presence of AZM at various concentrations and the relative RNA levels of PA3297 were determined by real time PCR. Indeed, the expression of PA3297 was induced by AZM (**Figure 5A**). However, overexpression of PA3297 in wild type PA14 did not further increase the bacterial tolerance to AZM (**Table 4**). We suspect that since the bacteria at exponential growth phase are highly resistant to macrolides and lincosamides, overexpression of PA3297 might not further increase the resistance significantly in the MIC test. Another possibility is that with the endogenous up regulation of PA3297, additional expression of PA3297 might be redundant.

**Table 4:** Bacterial susceptibilities to macrolides and lincosamides.

Strain	MIC (μg/ml)^a^			
	
	ERY	AZM	LIN	CLI
PA14/pMMB67EH	600	200	12800	12800
PA14/pMMB67-PA3297	600	200	12800	6400
PA14/pMMB67-*ermC*	2400	800	>12800	>12800
ΔPA3297/pMMB67EH	75	50	6400	3200
ΔPA3297/pMMB67-PA3297	300	200	12800	3200
ΔPA3297/pMMB67-*ermC*	2400	400	>12800	>12800


*^a^ERY, erythromycin; AZM, azithromycin; LIN, lincomycin; CLI, clindamycin.*

Köhler et al. (2007) previously demonstrated that ribosome is the only target of AZM in bacteria. To test whether the induction of PA3297 is caused by the interaction between AZM and ribosome, we performed the ribosomal protection assay by overexpressing ErmC, a 23S rRNA methylase that blocks the binding of macrolide antibiotics to the NPET (Köhler et al., 2007; Lawrence et al., 2008). In the presence of 10 μg/ml AZM, the growth speed of the ErmC overexpressing strain was similar with that of the wild type strain containing an empty vector. However, overexpression of ErmC abolished the induction of PA3297 by AZM (**Figure 5B**). In addition, antibiotics in the other categories, including ciprofloxacin, tobramycin, and carbenicillin did not affect the expression level of PA3297 (**Supplementary Figure S2**). These results suggest that the expression of PA3297 is regulated in response to AZM-mediated ribosome stalling.

### Ribosome Protection Rescues the ΔPA3297 Mutant from AZM-Mediated Hyperlethality

**FIGURE 6:**
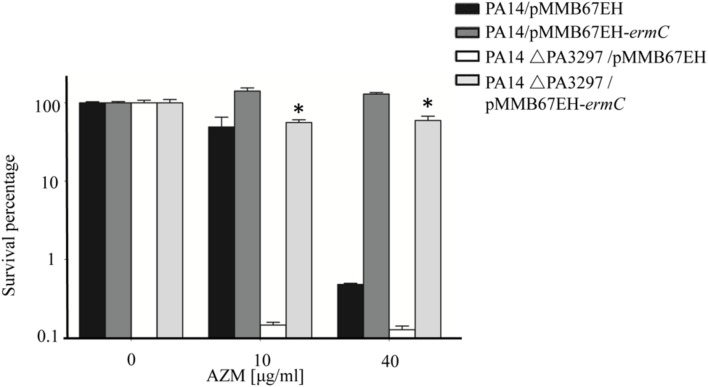
**Effects of Ribosome protection on the AZM mediated killing of the ΔPA3297 mutant.** Wild type PA14 or the ΔPA3297 mutant harboring pMMB67EH or pMMB67EH-*ermC* was grown to an OD_600_ of 0.5 when 1 mM IPTG was added to the medium. When the OD_600_ reached 2.0, the bacteria were treated without or with 10 or 40 μg/ml AZM for 20 h at 37°C. The numbers of live bacteria were determined by serial dilution and plating. ^∗^*p* < 0.05 compared to the ΔPA3297 mutant harboring pMMB67EH by student’s *t*-test.

So far, we have demonstrated that the expression of PA3297 is induced by AZM and that mutation of PA3297 renders *P. aeruginosa* hypersusceptible to AZM. These results suggest that PA3297 might play a role in counteracting the detrimental effects caused by the interaction between AZM and ribosome (Köhler et al., 2007; Gödeke et al., 2013). Thus, ribosome protection should be able to increase the tolerance of the ΔPA3297 mutant to AZM. Indeed, overexpression of ErmC increased both the growth speed and the survival rate of the ΔPA3297 mutant when treated with 10 or 40 μg/ml AZM (**Supplementary Figure S3**, **Figure 6**). In addition, overexpression of ErmC increased the MICs of both wild type PA14 and the ΔPA3297 mutant to AZM, erythromycin, lincomycin and clindamycin (**Table 4**). Mutation of PA3297 did not increase the bacterial susceptibility to a variety of other antibiotics (**Supplementary Table S1**). These results suggest a specific role of PA3297 in responding to lincosamides and macrolides.

### Deficiency in PA3297 Compromises rRNA Processing in the Presence of AZM

**FIGURE 7:**
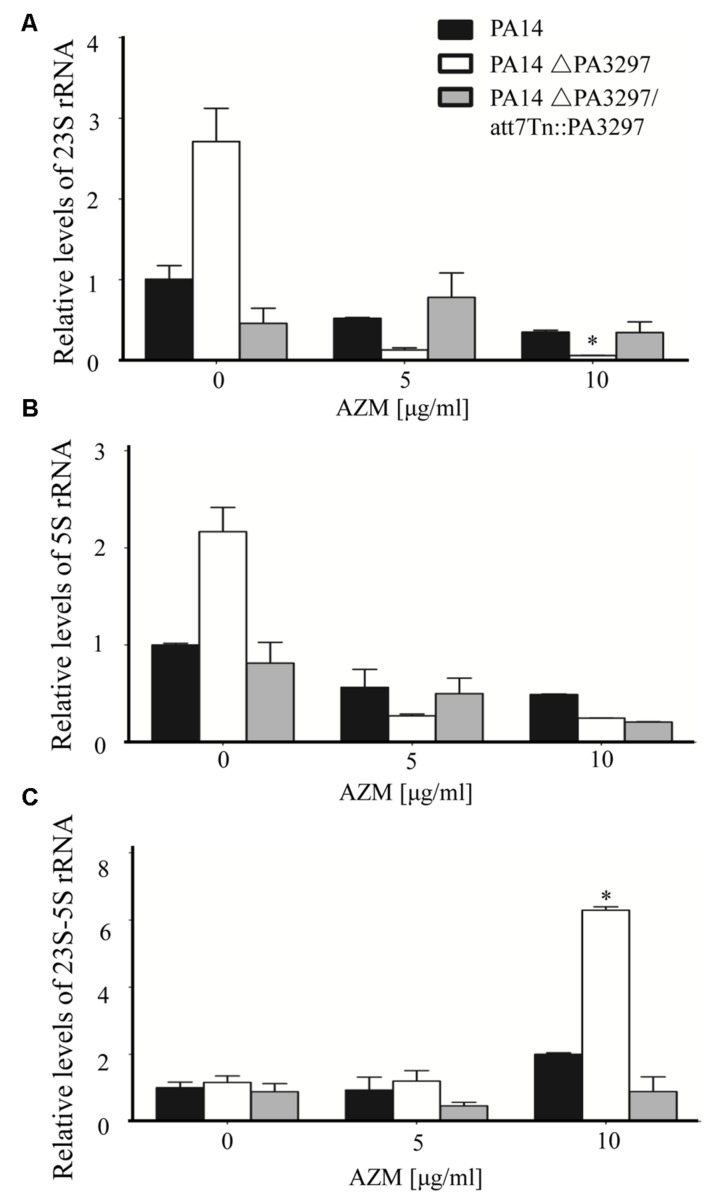
**Effects of AZM on the processing of rRNA.** PA14, the ΔPA3297 mutant and the complemented strain were grown at 37°C in the absence of AZM. When the OD_600_ reached 0.3, AZM (0, 5, and 10 μg/ml) was added. Total RNA was harvested when the OD_600_ reached 2.0. The levels of total 23S **(A)**, 5S **(B)**, and unprocessed 23S-5S rRNA **(C)** were determined by real time PCR. The mRNA levels of PA1769 were used as an internal control. ^∗^*p* < 0.05 compared to PA14 by student’s *t*-test.

Studies in *E. coli* demonstrated that the DExD/H box play crucial roles in rRNA processing (Iost et al., 2013; Linder and Fuller-Pace, 2013). Therefore, we suspected that the up regulated PA3297 might participate in rRNA maturation, which facilitates ribosome biogenesis to compensate for AZM inactivated ribosome. The rRNA coding region in the chromosome of *P. aeruginosa* PA14 is shown in **Supplementary Figure S4**. To examine the processing of the rRNA transcript, we designed real-time PCR primers to analyze the total 23S and 5S rRNA levels as well as primers across the 23S and 5S rRNA coding region to analyze the level of unprocessed rRNA (**Supplementary Figure S4**). In wild type PA14, AZM at the concentrations of 5 and 10 μg/ml reduced the 23S and 5S rRNA levels (**Figures 7A,B**), and 10 μg/ml AZM slightly increased the unprocessed 23S-5S rRNA level (**Figure 7C**). In the absence of AZM, the total 23S and 5S rRNA levels were higher in the ΔPA3297 mutant than those in the wild type strain (**Figures 7A,B**), whereas the unprocessed 23S-5S levels were similar between the mutant and wild type strain. Interestingly, treatment with 10 μg/ml AZM resulted in a higher level of unprocessed 23S-5S rRNA in the ΔPA3297 mutant (**Figure 7C**), although its total 23S and 5S rRNA levels were lower than those in the wild type strain (**Figures 7A,B**).

Next, we calculated the percentages of unprocessed 23S-5S rRNA in wild type PA14 and the ΔPA3297 mutant with or without AZM treatment. Standard curves were generated to determine the amplification efficiencies of the primer pairs for the detection of total 23S, 5S and unprocessed 23S-5S rRNA levels in real time PCR (**Supplementary Figure S5**). Considering 5S rRNA is more prone to be lost during RNA purification, we calculated the ratio of unprocessed 23S-5S rRNA by dividing the levels of 23S-5S rRNA by those of 23S rRNA calibrated with the amplification efficiencies (**Table 5**). In the absence of AZM, the ratios of unprocessed 23S-5S rRNA were approximately 0.014 and 0.009% in wild type PA14 and the ΔPA3297 mutant, respectively. It seems that, without PA3297, the processing of 23S-5S rRNA is even more efficient. In the presence of 5 and 10 μg/ml AZM, the ratios of unprocessed 23S-5S rRNA rose to approximately 0.05 and 0.09% in wild type PA14, respectively. However, the ratios of unprocessed 23S-5S rRNA in the ΔPA3297 mutant were 0.33 and 1.61%, which were approximately 5- and 18-fold higher than those in PA14 under the same condition (**Table 5**).

**Table 5:** The ratio of unprocessed 23S-5S rRNA in total 23S rRNA (%).

Strain	AZM (μg/ml)		
	
	0	5	10
PA14	0.014	0.051	0.089
ΔPA3297	0.009	0.335	1.608


*The unprocessed 23S-5S rRNA ratios were calculated according to the equation: Ratio = (1+e_*23S*_)^*C*^_*t*_^*(23S)*^/(1+e_*23S-5S*_)^*C*^_*t*_^*(23S-5S)*^; e: Amplification efficiency. C_*t*_: Cycles when fluorescence intensity reaches detection threshold.*

Since, the growth speed of the ΔPA3297 mutant in the presence of 10 μg/ml AZM was similar as that of the wild type PA14 in the presence of 40 μg/ml AZM (**Supplementary Figure S3**), we compared the rRNAs levels of the two strains grown under the two conditions. The relative levels of 23S-5S rRNA, total 5S and 23S rRNA in the ΔPA3297 mutant were 150, 55, and 100% of those in the wild type PA14, respectively, indicating a similar rRNA processing status with the two different AZM concentrations. In combination, these results suggest that PA3297 might contribute to rRNA processing in response to AZM. And mutation of PA3297 might impair the biosynthesis of ribosome under AZM treatment, which renders the bacterium more susceptible to AZM.

## Discussion

By binding to 23S rRNA in the 50S subunit of bacterial ribosome, AZM blocks polypeptide elongation and diminishes the intracellular pools of aminoacyl-tRNAs (Tenson et al., 2003; Gödeke et al., 2013; Wilson, 2014). And it has been demonstrated that AZM reduces the expression of *gacA* and the small RNAs *rsmY* and *rsmZ*, as well as quorum sensing genes (Kai et al., 2009; Pérez-Martínez and Haas, 2011). And the stationary phase killing by sub-MIC AZM was demonstrated to be correlated with increased outer membrane permeability (Imamura et al., 2005). Here, we found that mutation of PA3297 intensified the AZM mediated inhibitory effects on pyocyanin production and swarming motility of *P. aeruginosa.* In addition, the PA3297 mutant is more susceptible to oxidative stress in the presence of AZM. During infection, host generated reactive oxygen species (ROS) is an important bacterial killing mechanism. Therefore, inhibition of PA3297 together with the treatment with AZM, might render the bacteria more susceptible to host killing.

The MIC of the ΔPA3297 mutant is a quarter of that of the wild type strain. Consistently, the growth speed of the ΔPA3297 mutant in the presence of 10 μg/ml AZM was similar as that of the wild type strain in the presence of 40 μg/ml AZM (**Supplementary Figure S3**). However, in the stationary phase cell killing assay, the survival rate of the ΔPA3297 mutant treated with 10 μg/ml AZM was approximately 20% of that of the wild type strain treated with 40 μg/ml AZM (**Figure 6**). Therefore, PA3297 might play a more important role in the survival of stationary phase cells under AZM treatment.

Macrolides and lincosamides bind to the 50S of ribosome and block the NPET (Tenson et al., 2003). In our experiments, mutation in PA3297 did not alter the bacterial resistance to other antibiotics, including those binding to 30S or other parts of 50S ribosome. In addition, the expression of PA3297 was upregulated by AZM, which was abolished by ribosome protection. Therefore, PA3297 might specifically play a role in bacterial response to macrolides and lincosamides.

PA3297 is also named HrpA, both in PAO1 and PA14 (Winsor et al., 2016). According to the Profiles from GEO Expression Database at NCBI, the expression level of PA3297 was higher in biofilm than that in planktonic cells (Anderson et al., 2008). Isolates from CF lungs displayed higher expression levels of PA3297 levels than PAO1 (Son et al., 2007; Bielecki et al., 2013). And artificial medium that mimics CF lung sputum could increase the expression level of PA3297 slightly (Fung et al., 2010). In addition, increase of PA3297 expression was also observed in antibiotic-resistant small colony variants (Wei et al., 2011). However, the expression level of PA3297 showed a significant decrease in response to airway epithelia or low oxygen conditions (Alvarez-Ortega and Harwood, 2007; Chugani and Greenberg, 2007). These results indicate that the expression of PA3297 is regulated in response to various environmental stesses.

PA3297 is predicted to locate at the cytoplasmic membrane, with a molecular weight of 149.8 kDa (Winsor et al., 2016). It belongs to the DExD/H box helicase family. Members of this family have been found to play crucial roles in RNA metabolism and gene regulation (Linder and Jankowsky, 2011; Iost et al., 2013; Kaberdin and Bläsi, 2013; Linder and Fuller-Pace, 2013; Putnam and Jankowsky, 2013). According to NCBI protein blast, there are 81 homologous proteins with identities of 78% or more in other microorganisms. The homolog of PA3297 in *Borrelia burgdorferi* was found to be required for mouse infectivity and tick transmission and involved global gene regulation (Salman-Dilgimen et al., 2011; Owttrim, 2013). Another homolog in *E. coli* was found to be involved in fimbrial biogenesis (Koo et al., 2004). The identities they shared with PA3297 are 34 and 49%, respectively. Both of them possess the conserved residues in motif I, motif II, and motif III. Meanwhile, they are also involved in RNA processing. In *Listeria monocytogenes*, it has been reported that defect of a DExD-box RNA helicase, Lmo1722, reduced the maturation of 23S RNA (Bareclev et al., 2014) at low temperatures.

In *E. coli*, the primary transcript rRNA is cleaved by RNase III, yielding precursors of the 16S rRNA (17S rRNA), 23S rRNA, and 5S rRNA (9S rRNA; Shajani et al., 2011). Mutation of RNase III led to slower growth rate, reduction in cell viability and protein synthesis rates in the presence of AZM (Silvers and Champney, 2005). In *E. coli*, under certain stress conditions, DEAD box helicase may substitute for RhlB in the degradosome, such as CsdA under cold shock conditions (Prud’homme-Généreux et al., 2004). We found that mutation of PA3297 increased the percentage of unprocessed 23S-5S rRNA in the presence of AZM, which indicates that the cleaving function of RNase III might be impaired under the stress caused by AZM. Therefore, PA3297 might assist RNase III in rRNA processing in the presence of AZM. In addition, although the growth speed and rRNA processing in the ΔPA3297 mutant treated with 10 μg/ml AZM were similar to those in the wild type strain treated with 40 μg/ml AZM (**Supplementary Figure S3**), the survival rate of the ΔPA3297 mutant was lower than that of the wild type strain under those conditions. Thus, PA3297 might play other roles in counteracting the effects of AZM.

Overall, mutation of PA3297 renders *P. aeruginosa* more susceptible to AZM mediated inhibition on virulence factors and killing effect. Therefore, targeting the regulatory pathway or the function of PA3297 might further increase the beneficial effects of AZM in the treatment of chronic *P. aeruginosa* infections.

## Author Contributions

Conceived and designed the experiments: WW, SJ, HT, ZC. Performed the experiments: HT, LZ, YW, RC, FZ, YJ. Analyzed the data: HT, LZ, SJ, WW. Wrote the paper: HT, SJ, WW.

## Conflict of Interest Statement

The authors declare that the research was conducted in the absence of any commercial or financial relationships that could be construed as a potential conflict of interest.
