# Supplementary material for: PA3297 Counteracts Antimicrobial Effects of Azithromycin in Pseudomonas aeruginosa
Source: Front Microbiol. 2016 Mar 16;7:317. doi: 10.3389/fmicb.2016.00317 (PMC4792872; doi:10.3389/fmicb.2016.00317)
Supplement: Supplementary file 1 [file Table_1.DOC]

**TABLE S1**. Bacterial susceptibilities to antibiotics

| Strain | MIC (μg/ml)^a^ | | | | | | | |
| --- | --- | --- | --- | --- | --- | --- | --- | --- |
|  | CIP | CAR | MEM | TET | TOB | KAN | CHL | PMB |
| PA14 | 0.25 | 110 | 1.25 | 25 | 1.125 | 150 | 100 | 4 |
| ΔPA3297 | 0.25 | 110 | 1.25 | 25 | 2.25 | 150 | 100 | 4 |

^a^ CIP, ciprofloxacin; CAR, carbenicillin; MEM, meropenem; TET, tetracycline; TOB, tobramycin; KAN, kanamycin; CHL, chloramphenicol; PMB, polymyxin B.
