# Supplementary material for: PA3297 Counteracts Antimicrobial Effects of Azithromycin in Pseudomonas aeruginosa
Source: Front Microbiol. 2016 Mar 16;7:317. doi: 10.3389/fmicb.2016.00317 (PMC4792872; doi:10.3389/fmicb.2016.00317)
Supplement: Supplementary file 2 [file Image_1.PDF]

FIG. S1

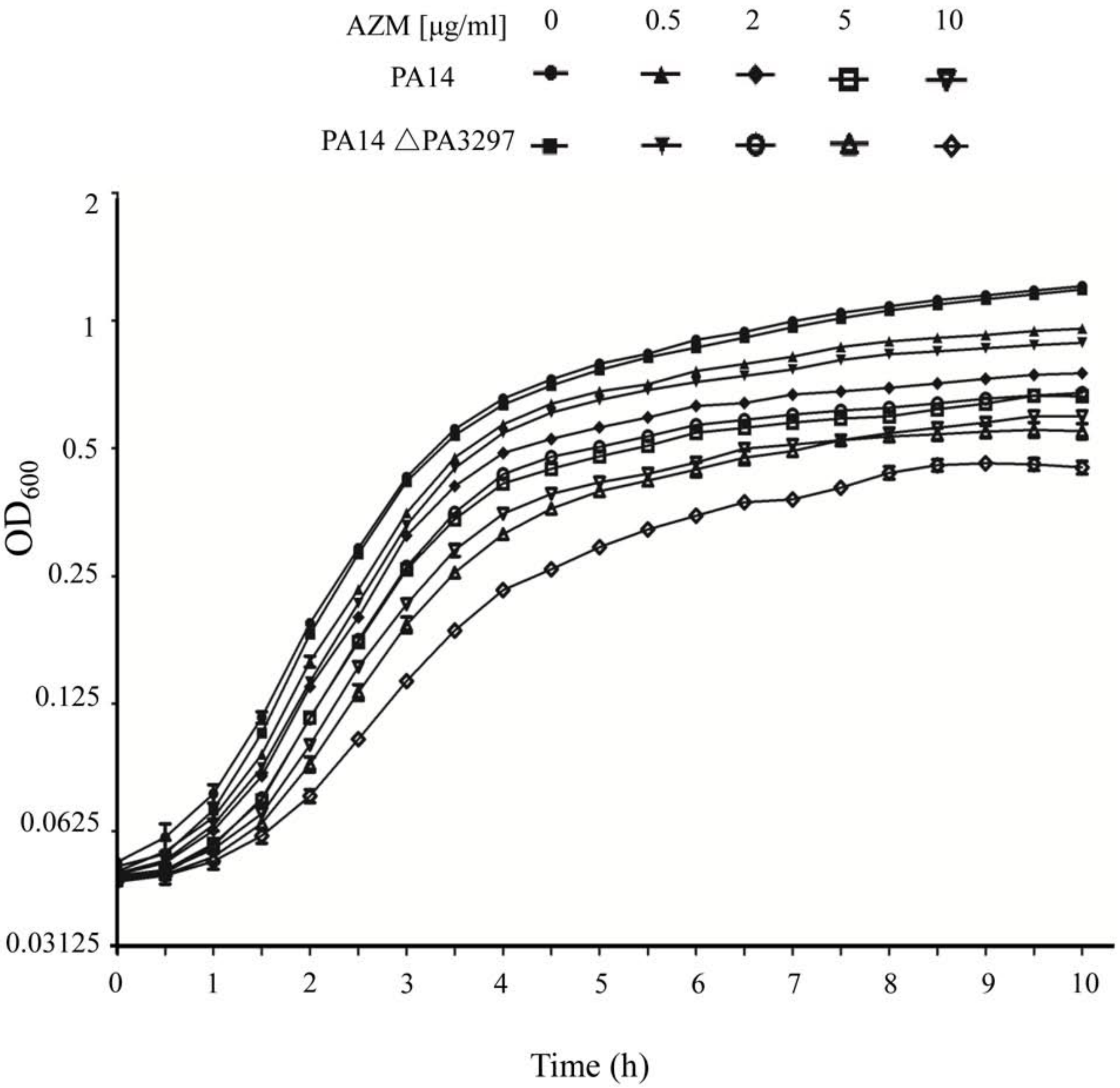

Fig. S1 Growth of wild type PA14 and the ΔPA3297 mutant in the absence or presence of AZM. Overnight cultures of PA14 and the ΔPA3297 mutant were diluted into fresh LB medium to reach an OD_{600} of 0.05. No AZM or AZM at the final concentrations of 0.5, 2, 5 and 10 μg/ml was added. The values of OD_{600} were measured every 30 minutes for 10 hours.
