# Supplementary material for: PA3297 Counteracts Antimicrobial Effects of Azithromycin in Pseudomonas aeruginosa
Source: Front Microbiol. 2016 Mar 16;7:317. doi: 10.3389/fmicb.2016.00317 (PMC4792872; doi:10.3389/fmicb.2016.00317)
Supplement: Supplementary file 3 [file Image_2.PDF]

FIG. S2

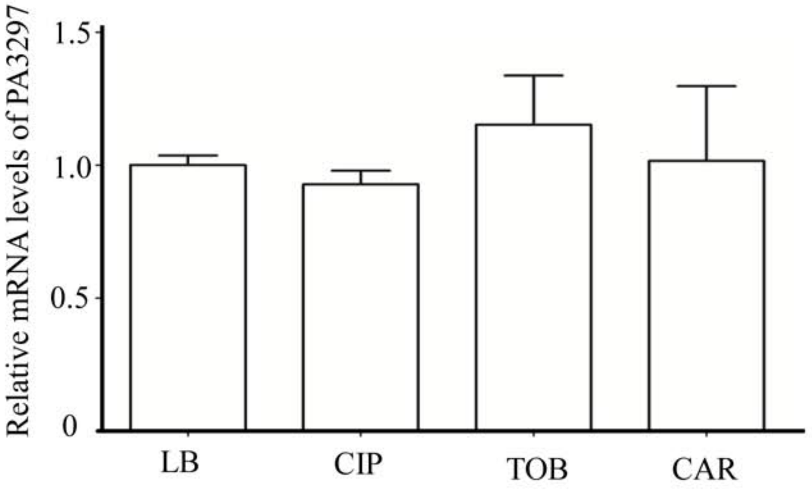

Fig. S2 Expression of PA3297 in the presence of indicated antibiotics. Wild type PA14 was grown to an OD_{600} of 0.3 in LB medium. Then 1/20 MIC of ciprofloxacin (CIP), tobramycin (TOB) or carbenicillin (CAR) was added to the medium . When the OD_{600} reached 2.0, total bacterial RNA was isolated and the mRNA levels of PA3297 were determined with real time PCR. The mRNA levels of PA1769 were used as an internal control.
