# Supplementary material for: PA3297 Counteracts Antimicrobial Effects of Azithromycin in Pseudomonas aeruginosa
Source: Front Microbiol. 2016 Mar 16;7:317. doi: 10.3389/fmicb.2016.00317 (PMC4792872; doi:10.3389/fmicb.2016.00317)
Supplement: Supplementary file 4 [file Image_3.PDF]

FIG. S3

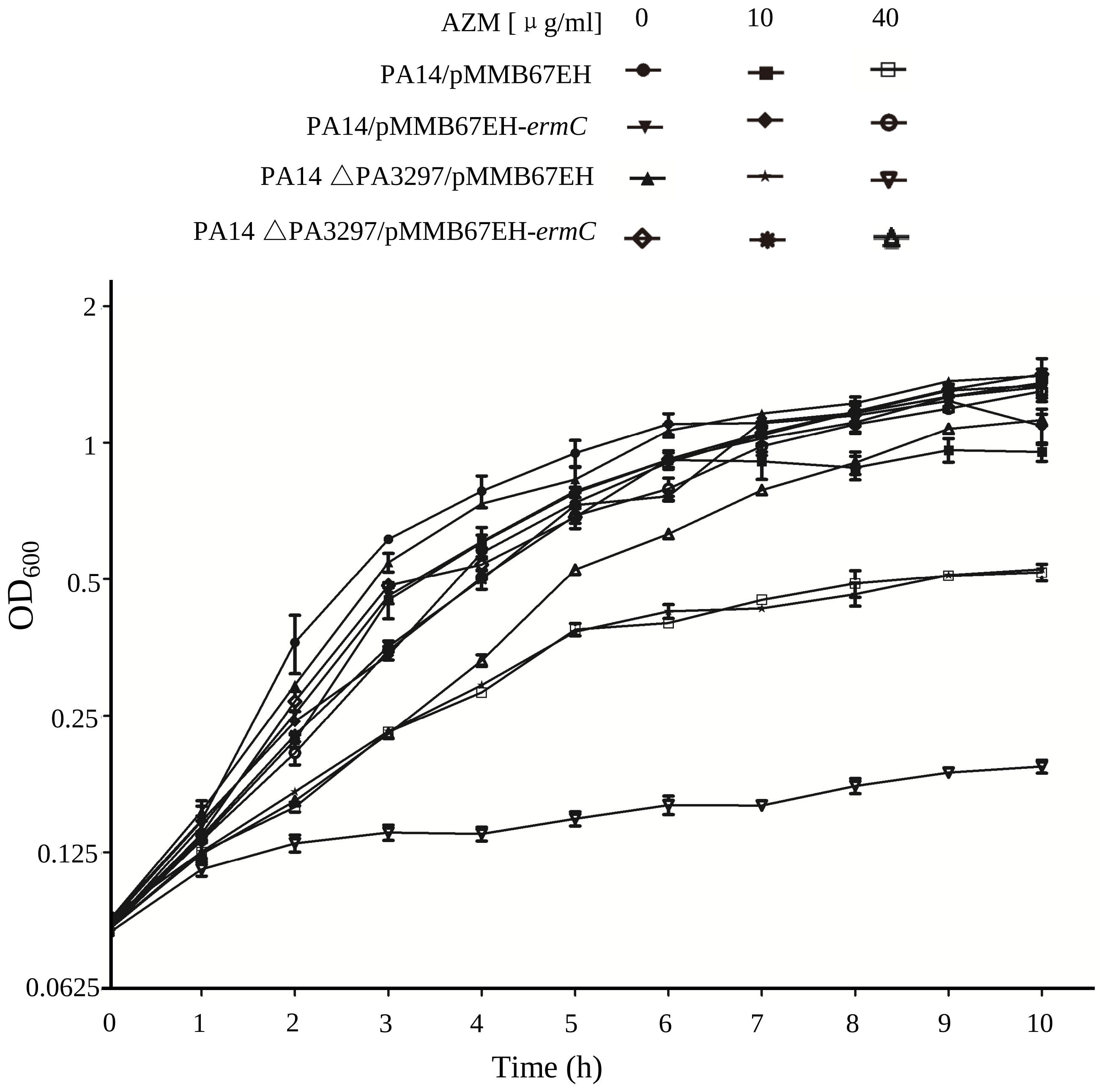

Fig. S3 Growth of wild type PA14 and the △PA3297 mutant harboring empty vector or the *ermC* overexpression plasmid in the absence or presence of AZM. Overnight bacterial cultures were diluted into fresh LB medium to the same OD_{600}. No AZM or AZM at the final concentrations of 10 and 40 μg/ml was added. The values of OD_{600} were measured every hour for 10 hours.
