# Supplementary material for: PA3297 Counteracts Antimicrobial Effects of Azithromycin in Pseudomonas aeruginosa
Source: Front Microbiol. 2016 Mar 16;7:317. doi: 10.3389/fmicb.2016.00317 (PMC4792872; doi:10.3389/fmicb.2016.00317)
Supplement: Supplementary file 5 [file Image_4.PDF]

FIG. S4

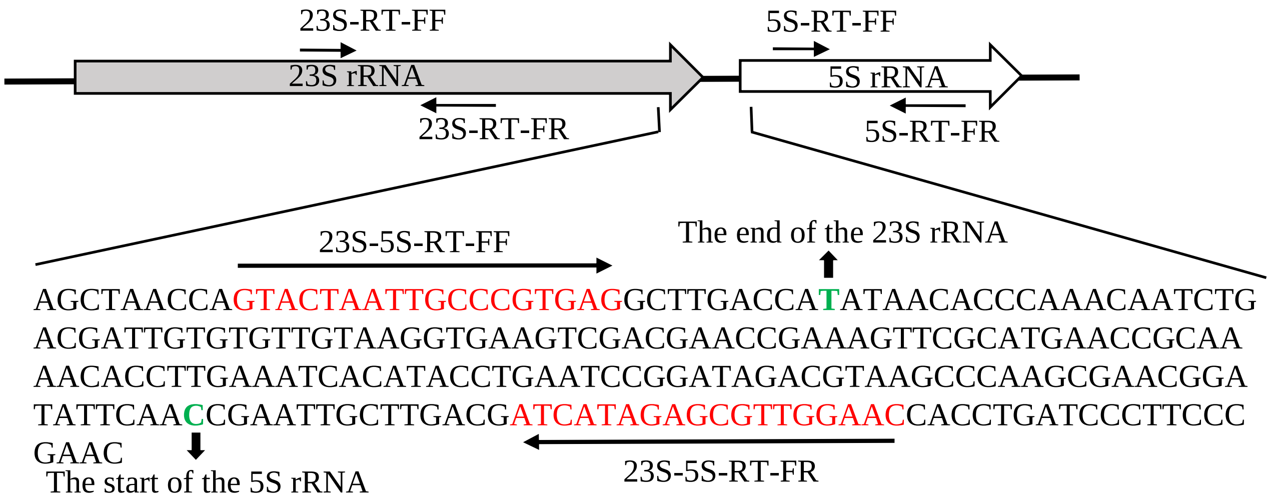

FIG. S4 Genetic organization of the 23S and 5S rRNA coding region of *P. aeruginosa* PA14. The arrows represent the locations and directions of the primers used in real time PCR. The sequences of the real time PCR primers for the detection of unprocessed 23-5S rRNA were shown in red. The end of the 23s rRNA and the start of the 5S rRNA coding regions were shown in green.
