# Supplementary material for: PA3297 Counteracts Antimicrobial Effects of Azithromycin in Pseudomonas aeruginosa
Source: Front Microbiol. 2016 Mar 16;7:317. doi: 10.3389/fmicb.2016.00317 (PMC4792872; doi:10.3389/fmicb.2016.00317)
Supplement: Supplementary file 6 [file Image_5.PDF]

FIG. S5

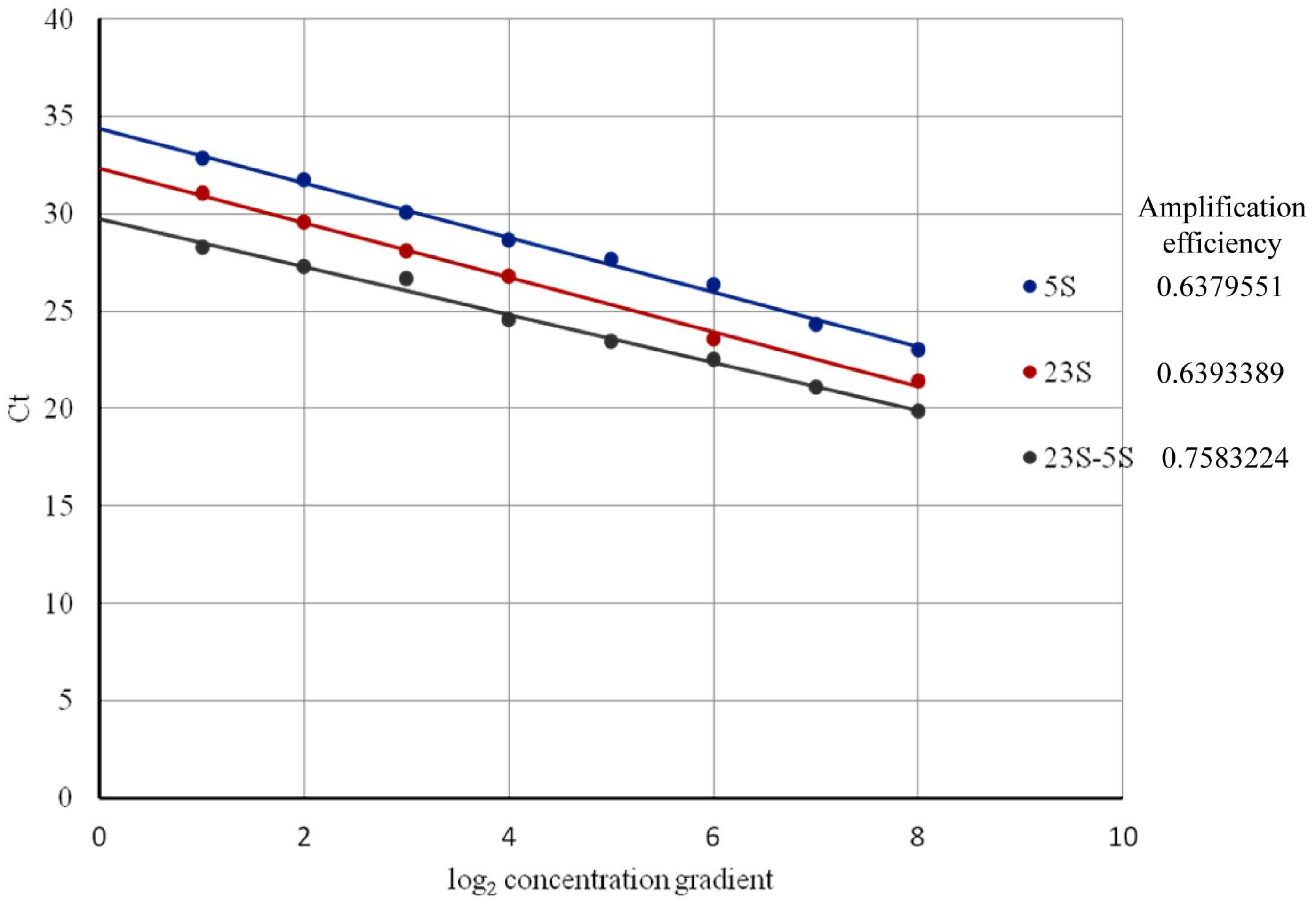

FIG. S5 The amplification efficiency. cDNA from PA14 wild type treated with 10 μg/ml AZM was serially diluted and used as template in real time PCR. The corresponding formula of fitted curve for 5S rRNA amplification is $y = -1.4047x + 34.398$, $R^2 = 0.9949$; for 23S rRNA: $y = -1.4023x + 32.329$, $R^2 = 0.9966$; for 23S-5S rRNA $y = -1.2282x + 29.726$, $R^2 = 0.9918$. The amplification efficiency (e) was calculated as: $e=2^{(-1/k)} -1$
